# Management equity incentives and corporate tax avoidance: Moderating role of the internal control

**DOI:** 10.3389/fpsyg.2023.1096674

**Published:** 2023-01-30

**Authors:** Xie Wenwu, Muhammad Usman Khurram, Lian Qing, Asia Rafiq

**Affiliations:** ^1^Institute of Digital Finance, Zhejiang University City College, Hangzhou, China; ^2^School of Business, Zhejiang University City College, Hangzhou, China; ^3^College of Economics, Zhejiang University, Hangzhou, China; ^4^Institute of Business Management Sciences, University of Agriculture, Faisalabad, Pakistan

**Keywords:** enterprise tax avoidance, management equity incentive, ownership type, internal controls, state-owned enterprise (SOE)

## Abstract

**Introduction:**

Under the modern enterprise system, the principal-agent relationship can cause a conflict of interest between the two power counterparts, thus affecting the degree of corporate tax avoidance. As a tool to align the interests of management and owners, management equity incentives can alleviate the conflict of interests brought about by the separation of powers and, therefore, may influence corporate tax avoidance.

**Objectives and methods:**

We examine the relationship between management equity incentives and corporate tax avoidance from both theoretical and empirical perspectives by using data from Chinese A-share listed companies from 2016 to 2020. Firstly, the effect of management equity incentives on tax avoidance is theoretically and normatively analyzed. Secondly, examine the effectiveness of moderating the effect of internal control and distinguishing the ownership of enterprises’ nature through regression analysis.

**Results:**

(1) There is a positive relationship between management equity incentives and corporate tax avoidance which means, more the stock incentive offered to executives, the more likely corporations are to pursue tax avoidance strategies aggressively. (2) Internal control deficiencies enhance the positive relationship between equity incentives and enterprise tax avoidance behavior. Therefore, in Chinese enterprises, the lack of an internal control system and the failure of internal control measures are prevalent, and such loopholes can intensify the tax avoidance behavior that arises when executives are subject to equity incentives. (3) The influence of management equity incentives on enterprise tax avoidance behavior is greater in state-owned (SOE) than private enterprises. State-owned enterprises are more likely to increase enterprise tax avoidance behavior when management is subject to equity incentives for reasons such as strict performance requirements, lower regulatory oversight, and less interference from negative information. Finally, our findings have significant implications for policymakers/regulators, public companies, investors, standard setters, managerial labor markets, and the welfare of the overall economy.

## 1. Introduction

China’s socialist market economic system has improved since reform and opening up, and the current taxation system has been steadily applied in depth. Taxation has promoted the modernization of China and the improvement of comprehensive national power and plays an irreplaceable and huge role in today’s society. Since the corporate income tax in China reached RMB 4.2 trillion in 2021, it is a substantial source of tax revenue.^1^ The Chinese government has also implemented many enterprises taxation changes since 1979, which required major SOE 55%, small SOEs 10–55% and private corporations’ 35% income taxes (Cai and Liu, 2009). However, taxation will directly lead to the reduction of taxpayers’ wealth, and because China’s taxation law system is improving and the intensity of supervision is getting higher, tax evasion has a high risk, which makes taxpayers start to worry whether they will be punished by taxation authorities and turn to tax avoidance^2^ to reduce their tax burden. The significance of corporate tax as a source of tax collection has therefore sparked worries about corporate tax avoidance due to its financial effect. Many countries’ public budgets have dropped as a consequence of a substantial rise in corporate tax avoidance during the previous decade. An estimated US$500–650 billion worldwide is lost to tax avoidance every year, with low- and lower-middle-income nations accounting for one-third of the total. According to Dyreng et al. (2017), corporate tax avoidance has sharply grown during the last 25 years. According to Forbes (2017), the annual cost of tax evasion in the United States is projected to be over $200 billion. Almost 73% of Fortune 500 corporations have one or more subsidiaries in nations that are tax havens.^3^ Government revenue is severely imbalanced as a result of these tax tactics. In recent years, Chinese enterprises’ tax avoidance activities have become more intense. On the other hand, the law enforcement of taxation authorities in the collection and management work objectively provides conditions for enterprises to avoid taxation. According to data from the State Administration of Taxation, anti-tax avoidance in China generated just RMB 460 million in tax income in 2005, but in 2015, anti-tax avoidance contributed RMB 58 billion in tax revenue, a 126-fold increase in 10 years. The above information reflects the success of domestic anti-tax avoidance work and the current prevalence and severity of tax avoidance in China.

Management incentives are an important topic for research on corporate governance. Currently, the corporate governance environment in China is not sound enough, leading to serious agency problems between owners and managers of many companies and even the corrupt behavior of individual executives pursuing personal interests while ignoring owners’ interests. Although there has been a lot of study on the influence of managerial incentives on corporate governance in the academic community, there has been little research on the impact of tax avoidance (Desai and Dharmapala, 2006; Rego and Wilson, 2012; Armstrong et al., 2015; Khan et al., 2017, Khan et al., 2019). The research on this issue can make up for the lack of research on tax-related issues of management equity incentives and provide theoretical guidance to further improve the management of equity incentives of listed companies. Simultaneously, most international researchers examine relevant problems using data from publicly traded corporations in Europe and the United States, most of which are privately held. Therefore, most foreign scholars do not consider the influence of company ownership when studying the link between management equity incentives and corporate tax avoidance. State-owned firms account for a major part of listed companies in China, and diverse ownership will result in various business models and attitudes, altering managers’ decision-making behavior (Richardson et al., 2016; Li et al., 2017; Wang et al., 2021). The empirical investigation of the impact of management equity incentives on corporate tax avoidance is of great theoretical value in this work, considering China’s current situation.

Compared with developed countries, China only started the pilot practice of equity incentive since the late 1990s, and only started to apply equity incentive in listed companies since 2005 when the share split reform was promoted, so the equity incentive started late, and the proportion is small. The study of the managerial equity incentive of listed firms in China is crucial for developing tax avoidance theory and enhancing corporate governance. It is emphasized that the study of corporate tax avoidance from the perspective of management equity incentive is conducive to the continuous standardization of incentive system and unreasonable incentives, reasonable tax planning and maximization of benefits. At the same time, it provides more reference materials for the tax supervision of China’s administrative organs, hoping to improve the efficiency of taxation supervision and management of China’s taxation agencies, and the symptoms of taxation loss can be alleviated. The decision to avoid taxes is influenced by moral hazard, tax-planning expenses, and the possibility to boost earnings. On the other hand, internal control is a crucial tool for mitigating risk and preventing corruption (Gong et al., 2021; Wang et al., 2022), and strong internal control enhances the compliance and efficacy of operations. However, internal control also refers to middle-level management control, bottom-level operational control, and top-level internal governance. The internal control system’s overall design is the responsibility of the board of directors, and its execution is the responsibility of executives and other staff members. Government agencies in China support the internal control system of listed firms, and the system is effectively developed; nonetheless, the outcome is unsatisfactory (Zhang et al., 2020). Executives and staff members are human resources that must be inspired to execute internal controls. Some researchers have conducted comparative studies, such as Henry et al. (2011) found that increasing executive salary can increase the efficacy of internal control. According to Balsam et al. (2014), applying management equity incentives improves the quality of internal control. Guo et al. (2016) discovered that cash profit sharing, union relations rules, retirement benefits, employee ownership and engagement and health and safety initiatives had a substantial influence on the quality of internal controls. According to Zhang et al. (2020), the strength of non-executive equity incentives can lower the risk of internal control vulnerabilities while also improving internal control efficacy.

Therefore, our findings contribute to the literature on executive compensation incentives, internal control deficiencies, and corporate tax avoidance. The main findings are as follows; we empirically analyze the relationship between management equity incentive and corporate tax avoidance by using Chinese A-share listed companies from 2016 to 2020. The results show that (1) There is a positive association between management equity incentive and enterprise tax avoidance behavior consistent with prior studies (Phillips, 2003; Robinson et al., 2010; Rego and Wilson, 2012; Gaertner, 2014; Powers et al., 2016; Khan et al., 2019); the more the stock incentive offered to executives, the more likely corporations are to pursue tax avoidance strategies aggressively. (2) Internal control deficiencies enhance the positive relationship between equity incentive and enterprise tax avoidance behavior. Therefore, in Chinese enterprises, the lack of an internal control system and the failure of internal control measures are prevalent, and such loopholes can intensify the tax avoidance behavior that arises when executives are subject to equity incentive. (3) The influence of management equity incentive on enterprise tax avoidance behavior is greater in state-owned firms than in private enterprises. State-owned enterprises are more likely to increase enterprise tax avoidance behavior when management is subject to equity incentive for reasons such as strict performance requirements, lower regulatory oversight, and less interference from negative information.

The remaining sections of the study are structured as follows: section “2. Literature review and hypothesis development” comprehensively reviews the relevant literature and development of the hypothesis, while section “3. Methodology” elaborates on the methodology, which includes primary data collection sources and the definition of the variables, model construction of the study. Section “4. Empirical analysis” determines the empirical results based on regression analyses with various robustness tests. Section “5. Conclusion and policy recommendations” provides the conclusion of the study with policy recommendations.

## 2. Literature review and hypothesis development

The contemporary business system’s separation of ownership and management can lead to a conflict of interest between the owner and the actual operator, thus creating a principal-agent relationship problem (Wang and Yao, 2021). At this time, the owner, in order to make himself obtain greater benefits and increase the value of his company, usually takes appropriate measures to motivate the manager so that the two are in the same direction of interests, and then motivates the manager to use effective tax avoidance measures to obtain greater benefits for the company. In other words, the incentive for executives becomes an important tool to reduce the conflict between the two (Liu et al., 2010). As a tool to make the interests of management and owners converge, equity incentive can alleviate the conflict of interests caused by the separation of powers and reduce agency costs (Dyreng et al., 2010).

Tax is one of the important components of enterprise cost, which directly leads to cash flow outflow (Huang et al., 2018). The level of tax burden of enterprises is also related to the competitiveness of enterprise industry, so in order to reduce enterprise cost, improve enterprise cash flow and enhance enterprise competitiveness enterprises are likely to carry out tax avoidance (Hanlon and Heitzman, 2010). Tax planning (or optimization) is one of the necessary tools to ensure that the enterprise can survive in the fierce market competition (Palan, 2020), and in China, the accounting system design is not consistent with the provisions of tax law. That is to say, enterprises can achieve surplus management by adjusting non-taxable items to reduce corporate taxes without affecting their accounting revenue (Henry, 2018; Balakrishnan et al., 2019). In addition, in the specific tax operation practice, the existing tax legal provisions and the legal system itself are not perfect, and various insurmountable problems and loopholes still exist, and these loopholes become the natural conditions for enterprises to carry out tax avoidance (Crocker and Slemrod, 2005). However, although aggressive tax avoidance can reduce the tax burden of enterprises, it will not only erode our tax revenue and affect the regulatory role of national taxation, but also enhance the legal risk of enterprises (Chen et al., 2020). As the reform of the taxation and legal systems continues to deepen and the taxation supervision continues to increase, higher requirements are put forward for enterprises’ risk control ability in taxation. Moreover, in some radical tax avoidance process, it is often difficult for some enterprises to avoid touching the bottom line of the law, making it difficult to control the enterprise’s tax avoidance risk (Sun and Wang, 2018).

Equity incentive can link company performance with corporate personal wealth because the agency theory can enhance the consistency of the interests of managers and shareholders and reduce the agency conflict between the two (Wang and Yao, 2021). How exactly equity incentive specifically affects corporate tax management? Some scholars argue that the higher the equity incentive of executives, the stronger their willingness to avoid taxes (Liu et al., 2010). Lv and Li (2012) argues that on this basis, this effect will be significantly weakened if there is a greater external risk. However, some scholars’ studies have also come up with different results. Executive incentives are adversely connected with the degree of company tax evasion, according to Chen and Tang (2012), and this relationship is particularly prominent in poorly managed organizations. The poorer the quality of accounting information disclosure, the bigger the company’s tax avoidance. The unfavorable association between tax avoidance and the quality of accounting information disclosure can be mitigated by increasing executives’ remuneration. On the basis of Desai and Dharmapala (2006), because the shelter of tax revenue and the potential tax avoidance income of managers are complimentary, raising the level of equity incentive may lower the amount of tax avoidance. Further studies, adding tax rate volatility, Armstrong et al. (2015) find a positive correlation between compensation for equity incentive and tax activism, while Peng (2017) finds that tax rate volatility results in a U-shaped relationship between equity incentive and corporate tax avoidance. In addition, differentiating the nature of ownership, it is found that state-controlled enterprises reduce corporate tax avoidance while privately held and foreign-owned enterprises increase corporate tax avoidance when managerial incentive compensation is increased (Zhou and Hu, 2021).

Therefore, how can we restrain corporate tax avoidance and regulate corporate tax behavior? Under the modern enterprise system, executives often become the core of corporate tax avoidance decisions. Then, it is essential to incorporate the study of corporate tax avoidance behavior into the framework of the principal-agent problem between owners and management. Desai and Dharmapala (2006) show that a higher equity incentive helps to align the incentives of principals, and as the share of managerial equity incentive increases, the incentive is more likely to be higher. As the share of management equity incentive increases, it makes management more inclined to work to maximize the interests of all shareholders through financial arrangements to save tax burden (Liu et al., 2010). At the same time, the agency view of tax avoidance thinks that tax avoidance tends to deepen the firm’s internal and external information asymmetry, which affects the firm’s value (Desai and Dharmapala, 2006). The degree of tax avoidance of the firm is significantly and positively related to inefficient investment, and tax avoidance reduces the efficiency of the firm’s investment (Sun and Wang, 2018; Hasan et al., 2022). That is to say, tax avoidance may increase the business risks faced by enterprises on the one hand and may also negatively affect enterprises’ investment efficiency and value.

At present, a large proportion of listed companies in some developed countries have adopted the management equity incentive approach. Incentives to management provide long-term incentives to improve performance by creating a corresponding link between higher pay performance sensitivity and lower taxes (Minnick and Noga, 2010). However, equity incentive, as a system of share-based compensation, is bound to impact corporate tax liabilities and corporate net income and shareholder interests (Lu and Yang, 2021). Management equity incentive affect management behavior, and tax avoidance activities are one of the management behaviors, i.e., management equity incentive affects corporate tax avoidance decisions. Management is further driven to seek corporate tax avoidance with the owners to maximize their benefits by receiving taxable equity benefits (Duru et al., 2012). Therefore, it is critical to investigate the relationship between equity incentive and corporate tax evasion as a study issue.

### 2.1. Research hypothesis

According to the incentive compatibility principle of principal-agent theory, if the management of a firm is to pursue the firm’s long-term interests as much as the owners, then it can only be motivated to have the expectation of long-term gains. According to Desai and Dharmapala (2006), a higher incentive level encourages proprietors, agents, and managers to be more radical in improving the firm’s profitability through tax avoidance. Managerial incentives, especially equity incentive, are the more important management system in modern management. In business, there may be agency problems between owners and operators due to the uneven distribution of benefits, which are often difficult to solve before equity incentive. The equity incentive can make the manager get a certain number of shares of the company, so that the company’s development is closely related to the manager’s interests, and the manager and the owner can reach a certain degree of consensus. Thus, after the managers receive the equity incentive, they can likely arrange tax avoidance measures to achieve the purpose of tax savings and increase corporate income. It leads to the first hypothesis of this paper:

**Hypothesis 1:** There is a positive link between management equity incentives and corporate tax avoidance.

Among the many corporate governance factors, there are more and more factors that can have an impact on tax risk. Internal control, as one of the corporate governance mechanisms, plays an irreplaceable role in corporate activities when uncertainties caused by macro and micro factors of corporate operations increase. Its main objectives are to provide reasonable assurance for the safety of corporate assets, to supervise the production and operation management in compliance with legal regulations, to ensure the truthfulness and completeness of financial reporting information disclosure, and to effectively improve the quality and efficiency of operation (Zhang, 2020). Henry et al. (2011) found that increasing executive package compensation can increase the efficacy of internal control. Balsam et al. (2014) reported that applying management equity incentives improves the quality of internal control. According to Zhang et al. (2020), the strength of non-executive equity incentives can lower the risk of internal control vulnerabilities while also improving internal control efficacy. Although the senior management makes the tax avoidance decision of the enterprise, it cannot do everything personally in the implementation process and requires the cooperation of all departments within the enterprise to carry out the whole process of production and operation, which results in internal coordination costs. If the internal control fails, it will cause the tax avoidance behavior of the enterprise to deviate from the expected goal (Zhang, 2021).

According to the report of “2018 White Paper on Internal Control of Chinese Listed Companies” by DIB Database, approximately 60% of listed companies’ internal control ratings in 2018 are concentrated at the B level, suggesting that the general degree of internal control of domestic listed companies is currently poor. In the internal control evaluation report for the reporting period of 2017, 456 organizations out of 34,875 listed corporations reported internal control violations (about 14% of the total number of enterprises in the internal control evaluation report, see Table 1 for details), compared with 512/2,864 in 2016, the overall indicator data decreased, but the number of listed companies in the category of “major and important internal control deficiencies” has been raising yearly, and the percentage of “major and important internal control deficiencies” in 2017 has increased by as much as 38% compared with the data in 2016.^4^

**TABLE 1:** Analysis of the status of disclosure of internal control deficiencies in listed companies.

Defect levels	Number of listed companies disclosing deficiencies	Percentage of total reporting companies^5^	Number of defects	Defect percentage
Major defects	65	2.016%	141	3.177%
Important defects	53	1.643%	85	1.915%
General defects	374	11.597%	4,212	94.908%
Total	456^6^	14.140%	4,438	100%

^5^A total of 3,225 listed companies disclosed their annual internal control evaluation reports during the 2017 reporting period.

^6^The reason why the total number of the three defect companies is greater than the total number of disclosure companies 456 is that some enterprises disclose more than one defect level of internal control defect information.

The number of serious internal control flaws has risen year after year in recent years, indicating that there is still considerable space for improvement in the quality of internal control in China’s publicly traded enterprises. Chinese government agencies support the internal control system of listed firms, and the system is effectively developed; nonetheless, the outcome is unsatisfactory (Zhang et al., 2020). In other words, most Chinese listed businesses’ internal control mechanisms are now insufficient, which leads to the second hypothesis of this research.

**Hypothesis 2:** Internal control plays an enhancing role in the positive relationship between corporate executive equity incentive on the degree of corporate tax avoidance.

In 2008, SASAC and the Ministry of Finance issued a notice on regulating the implementation of the equity incentive system in state-controlled listed companies, in which they once again emphasized the setting of performance targets and proposed that in the implementation of equity incentive in state-owned listed companies, performance targets should be set for both granting and exercising, and the performance targets should be set with the performance targets should be set in a forward-looking and challenging manner, and the completion of the performance evaluation index should be the condition for the implementation of the equity incentive. The stricter performance target setting makes enterprises have more incentives to adopt tax avoidance and other behaviors to meet the conditions of exercising or unlocking (Zhang et al., 2019). Meanwhile, the lack of supervision caused by the unique ownership structure of state-owned holding companies and the absence of owners may result in a decline in company internal control, resulting in a drop-in company supervision, making state-owned enterprises less transparent than private enterprises and making high tubes more likely to choose to increase tax avoidance through equity incentive (Wang et al., 2006; Li et al., 2017), thus providing more convenience for the management to implement tax avoidance behaviors. Through the tax avoidance by the management of state-owned enterprises, more cash flow is retained in the enterprise, which increases the after-tax profits of the enterprise and facilitates executives to accomplish the performance goals set in the equity incentive contracts (Zhang et al., 2019). Compared with state-controlled enterprises, private enterprises on the one hand have more flexibility in setting their performance targets, which can reduce the degree of corporate tax avoidance to a certain extent. On the other hand, for private firms, especially family firms where family members assume the role of managers, executives and firms generally have the same goals. Therefore, executives will focus more on maximizing the value of the enterprise, thus reducing their motivation to adopt tax avoidance and rent-seeking (Luo and Zeng, 2018).

In addition, SOEs and private firms react differently to negative information. For an enterprise, paying taxes can enhance its social image, while tax avoidance has more or less negative effects on the enterprise. SOEs are guaranteed by the reputation of the state and local government, while private enterprises are fully involved in the market competition and generally have a higher sense of crisis when there is negative news (Jie and Lihong, 2017). From the principal-agent perspective, private enterprises will be more strictly supervised by owners compared to state-owned enterprises, and executives may refrain from intervening in tax avoidance to reduce the generation of negative information due to their own interests (Zhang et al., 2019). As a result, the third hypothesis of this paper is introduced.

**Hypothesis 3:** Subject to other conditions, the nature of ownership of private enterprises has a weakening effect in the degree of influence of executive equity incentive on corporate tax avoidance relative to state-controlled enterprises.

## 3. Methodology

### 3.1. Sample and data source

The study sample for this paper is A-share companies listed in China from 2016 to 2020 and the necessary data is gathered from the CSMAR database, and the raw data are processed as follows: (1) ST companies and ST* companies are excluded. The reason is that ST companies and ST* companies have abnormal financial data in the sample observation period, which may cause extreme values and affect the empirical analysis. (2) Excluding companies with negative or zero accounting profit before tax. (3) Excluding the current income tax expense is negative or zero; the actual tax rate ETR is greater than 1 or less than 0 and cannot accurately obtain the taxable income amount. (4) Excluding companies with missing financial data. Finally, after screening, 3,341 A-share listed companies are obtained, with a total of 12,274 observation samples. All data analysis was performed by using Stata 16 statistical software.

### 3.2. Variables

#### 3.2.1. Explained variable

In this study, the current effective tax rate ETR is used as (Xie et al., 2023) an indicator of the corporate tax burden, and the effect of total corporate income tax expense and deferred income tax expense on the current effective tax rate is also considered:


(1) $$E\,T\,R=\frac{\mathrm{Income}\,\mathrm{tax}\,\mathrm{expense}-\mathrm{deferred}\,\mathrm{income}\,\mathrm{tax}\,\mathrm{expense}}{\begin{array}{c} \mathrm{Accounting}\,\mathrm{profit}\,\mathrm{before}\,\mathrm{tax} \end{array}}$$

Where deferred income tax expense = (Deferred income tax liabilities at the end of the period-deferred income tax liabilities at the beginning of period)-(Deferred income tax assets at the end of the period-deferred income tax assets at the beginning of period).

Considering that our government gives certain preferential policies to some enterprises in terms of applicable tax rates in order to support the development of certain enterprises, industries, or regions, which leads to differences in the corporate income tax rates applied by enterprises, which will have an impact on the measurement of corporate tax avoidance, the difference between the nominal corporate tax rate and its effective tax rate is used to measure the degree of corporate tax avoidance (Li et al., 2016; Tang et al., 2022). This method can better solve the situation that different enterprises apply different income tax rates. It is also more accurate than the effective tax rate method to measure the degree of corporate tax avoidance, and this paper also applies Li et al. (2016) approach:

The degree of corporate tax avoidance (TME) = nominal corporate tax rate (RATE)-current effective tax rate (ETR).

The greater the difference between the nominal tax rate and its effective tax rate, the greater the degree of tax avoidance of the enterprise.

#### 3.2.2. Explanatory variables

For measuring the level of management equity incentive, domestic research in China mainly selects the ratio of management shareholding to total company equity as a proxy variable for the level of management equity incentive directly. One way to evaluate the amount of executive stock options in foreign countries is the change in the value of executive stock and option when the share price of a company changes by 1%; the other is the change in the value of management stock and options when the value of the company changes by $1.

The company’s stock held by senior executives is the main manifestation of executive equity incentive, and senior executives can benefit from the stock held by senior executives. Compared with directly using the proportion of the number of senior executives in the total shares of the company to measure the degree of executive equity incentive, The index of the share of earnings from holding stocks in the total compensation structure of the executive can well measure the importance of equity incentive for the executive. Considering that equity incentive plans implemented by listed companies in China mainly announce the number of shares held by executives, this paper intends to learn from the methods of Desai and Dharmapala (2006), and from the perspective of executive compensation structure, compared with fixed compensation, the degree of equity incentive can be measured by whether equity incentive compensation has a significant incentive effect on senior executives. Based on the availability of data, the total compensation of the top three highest-paid executives was used to represent the executive compensation indicator. Among them, the management equity incentive is measured as part of the total compensation (excluding in-service expenses) and recorded as MRS, which is calculated as follows:


(2) Management⁢equity⁢incentive⁢(MRS)

=N⁢u⁢m⁢b⁢e⁢r⁢o⁢f⁢s⁢h⁢a⁢r⁢e⁢s⁢h⁢e⁢l⁢d⁢b⁢y⁢e⁢x⁢e⁢c⁢u⁢t⁢i⁢v⁢e⁢s×s⁢t⁢o⁢c⁢k⁢p⁢r⁢i⁢c⁢e×1%(Totalcompensationofthetopthreehighestpaidexecutives+numberofsharesheldbyexecutivesxstockpricex 1%)

A larger MRS calculated represents a higher degree of equity incentive for executives.

#### 3.2.3. Moderating variables

The prerequisite for quantifying internal control’s effectiveness is clarifying the corresponding evaluation criteria. This paper selects DIB internal control index to measure. This index was first released in 2011 and is jointly researched by Shenzhen DIB Company and Xiamen University, which has a certain authority. This index is based on the five elements of internal control, which can more objectively and comprehensively evaluate the effectiveness of the internal control system of various enterprises, applies to enterprises in different regions and industries, and can fully ensure the comparability between enterprises and themselves. The DIB, internal control index is used in this thesis to assess the efficacy of business internal control. In general, the DIB internal control index ranges from 0 to 1,000. The higher the index, the greater the quality of the enterprise’s internal control, and an index of 0 indicates the existence of major internal control flaws. In order to balance the numerical volume with other indicators and make the data results more accurate, the index is divided by 1,000; when the higher the index indicates, the better the quality of internal control.

#### 3.2.4. Control variables

The thesis draws on the model design of previous research on tax avoidance and selects the control variables that may affect corporate tax avoidance behavior as follows: enterprise size, enterprise growth capacity, non-interest tax shield, enterprise value, the shareholding ratio of the largest shareholder, enterprise age, while considering that scholars’ research results on corporate governance affect corporate tax avoidance decisions, holding both the positions of chairman and managing director is also a control variable. The definition of variables is shown in Table 2.

**TABLE 2:** Variable definition.

Variable type	Symbol	Variable	Measurement indicator
Explained variable	TME	The extent of corporate tax avoidance	Nominal corporate tax rate—current effective corporate tax rate
Explanatory variables	MRS	Management equity incentive	(Number of shares held by executives × stock price × 1%)/(Total compensation of the top three highest paid executives + number of shares held by executives × stock price × 1%)
Moderator variable	IC	Internal control effectiveness	DIB internal control index/1,000
Control variables	SIZE	Enterprise size	The natural logarithm of the total assets at the end of the period is taken
	GROWTH	Business growth capability	(Current period main business income -last period main business income)/last period main business income
	NDTS	Non-interest tax shield	(Accumulated depreciation of fixed assets + accumulated amortization of intangible assets)/ Total assets at the end of the period
	TQ	Enterprise value	Enterprise market value/asset replacement cost
	TOP1	Shareholding ratio of the largest shareholder	Number of shares held by the largest shareholder/Total number of shares at year-end
	DUAL	Two jobs at once	If the chairman and general manager are the same person, the value is 1; otherwise, the value is 0
	AGE	Enterprise age	The company’s listing years take the natural logarithm

In order to undertake a thorough investigation of the link between management equity incentive and corporate tax avoidance, this paper draws on previous research methods. It mainly adopts normative analysis and empirical research methods to conduct a theoretical and empirical study on the research topic.

To begin, the normative analysis method entails gathering, collating, analyzing, and researching existing literature on the impact of corporate tax avoidance and equity incentive on corporate tax avoidance, as well as conducting a theoretical analysis of the relationship between executive shareholding and the degree of corporate tax avoidance to establish the theoretical foundation for the study. This analysis is a prerequisite for empirical research.

Furthermore, using the empirical research approach, the hypothesis of the link between management equity incentive and corporate tax avoidance is provided, based on existing literature, under the premise that the real situation of listed businesses in China is completely integrated. Using the relevant data of China’s A-share listed enterprises from 2016 to 2020 as the sample, descriptive statistics, correlation tests, and regression analysis are conducted using Stata software to verify whether the hypothesis is correct and the robustness test is performed. Finally, the research conclusion is drawn, and recommendations are made. In other words, a quantitative analysis was conducted on the relationship between management equity incentive and corporate tax avoidance.

The following empirical models are created to evaluate the link between executive equity incentive and corporate tax avoidance derived from the aforementioned theoretical analysis: The relationship between the influence of management equity incentive on corporate tax avoidance behavior and firms with different ownership nature; Hypothesis 1 is tested by covering all samples. When hypothesis 3 is tested, state-controlled enterprises and private enterprises are included in the model separately to compare the relationship.


$$T\,M\,E_{i\,t}=\beta_{0}+\beta_{1}\,M\,R\,S_{i\,t}+\beta_{2}\,S\,I\,Z\,E_{i\,t}+\beta_{3}\,G\,R\,O\,W\,T\,H_{i\,t}+\beta_{4}\,N\,D\,T\,S_{i\,t}$$

(3) $$+\beta_{5}\,T\,Q_{i\,t}+\beta_{6}\,T\,O\,P\,1_{i\,t}+\beta_{7}\,D\,U\,A\,L_{i\,t}+\beta_{8}\,A\,G\,E_{i\,t}+\varepsilon_{i,t}$$

The effect of internal control in management equity incentive on corporate tax avoidance behavior; according to hypothesis 2, the internal control variable and the cross term are added to model 3.


$$T\,M\,E_{i\,t}=\beta_{0}+\beta_{1}\,M\,R\,S_{i\,t}+\beta_{2}\,I\,C_{i\,t}+\beta_{3}\,M\,R\,S*I\,C_{i\,t}+\beta_{4}\,S\,I\,Z\,E_{i\,t}$$

$$+\beta_{5}\,G\,R\,O\,W\,T\,H_{i\,t}+\beta_{6}\,N\,D\,T\,S_{i\,t}+\beta_{7}\,T\,Q_{i\,t}+\beta_{8}\,T\,O\,P\,1_{i\,t}$$

(4) $$+\beta_{9}\,D\,U\,A\,L_{i\,t}+\beta_{10}\,A\,G\,E_{i\,t}+\varepsilon_{i,t}$$

## 4. Empirical analysis

### 4.1. Descriptive statistics and correlation matrix

The descriptive statistics of the main variables shown in Table 3A indicate that enterprise size (SIZE) and enterprise value (TQ) are relatively large, while the fluctuations of the statistical indicators of the remaining variables are relatively small and within a reasonable range. The mean value of corporate tax avoidance (TME) is -0.024, and the standard deviation is 0.186, which indicates that tax avoidance is common among the listed companies in China’s A-share sector. The maximum value of management equity incentive (MRS) is 0.979, and the minimum value is close to 0, which is 0.000001, indicating that the degree of implementing equity incentive plan varies greatly among different listed companies in China, and there are companies in the observed sample that completely use equity incentive as the full compensation for executives, and there are also companies that do not implement equity incentive plan at all, indicating that there are huge differences in different companies’. This indicates that there is a huge difference in the attitudes and perceptions of different companies toward equity incentive. The maximum and minimum values of internal control effectiveness (IC) of enterprises are 0.941 and 0.00, respectively, indicating that the effectiveness of the implementation of internal control system varies among enterprises; the standard deviation is 0.130, explaining that there are differences in the effectiveness of the internal control system of enterprises. The mean value of SOE is 0.357, which indicates that the proportion of private enterprises in the study sample is about two-thirds, while the proportion of state-owned enterprises is about one-third, which is consistent with the actual situation in China.

**TABLE 3:** Descriptive statistics and correlation matrix.

Panel A: Descriptive statistics of main variables											
Variable	*N*		Mean		S.D.		Med		Min		Max
TME	12,274		−0.024		0.186		0.001		−5.232		1.779
MRS	12,274		0.339		0.393		0.055		0.000		0.979
SIZE	12,274		22.39		1.311		22.221		20.05		26.39
GROWTH	12,274		0.200		0.415		0.120		−0.466		2.923
NDTS	12,274		0.021		0.014		0.019		0.001		0.065
TQ	2,274		2.008		1.763		1.580		0.690		92.25
TOP1	12,274		0.339		0.145		0.320		0.029		0.891
DUAL	12,274		0.291		0.454		0.000		0.000		1.000
AGE	l12,274		2.173		0.815		2.303		0.000		3.434
IC	12,274		0.641		0.130		0.666		0.000		0.941
SOE	12,274		0.357		0.479		0.000		0.000		1.000
**Panel B: Descriptive statistics of enterprises with different ownership types**											
	**State-owned enterprises**						**Private enterprises**				
**Variable**	***N***		**Mean**		**S.D.**		***N***		**Mean**		**S.D.**
TME	3,988		−0.034		0.225		8,286		−0.019		0.164
MRS	3,988		0.048		0.157		8,286		0.479		0.395
SIZE	3,988		23.090		1.398		8,286		22.05		1.121
GROWTH	3,988		0.151		0.395		8,286		0.223		0.423
NDTS	3,988		0.024		0.015		8,286		0.020		0.013
TQ	3,988		1.718		1.471		8,286		2.147		1.872
TOP1	3,988		0.389		0.148		8,286		0.316		0.138
DUAL	3,988		0.093		0.291		8,286		0.386		0.487
AGE	3,981		2.649		0.660		8,188		1.696		0.936
**Panel C: Pearson correlation coefficient matrix**											
		**TME**		**MRS**	**SIZE**	**GROWTH**	**NDTS**	**TQ**	**TOP1**	**DUAL**	**AGE**
TME		1									
MRS		0.016		1							
SIZE		−0.031***		−0.231***	1						
GROWTH		0.031***		0.083***	0.055***	1					
NDTS		0.034***		−0.115***	−0.01	−0.074***	1				
TQ		0.048***		−0.020*	−0.216***	0.035***	0.005	1			
TOP1		0.009		−0.102***	0.003	−0.022**	0.040***	−0.020*	1		
DUAL		0.029***		0.112***	−0.111***	0.025**	−0.042***	0.058***	0.068***	1	
AGE		−0.040***		−0.377***	0.470***	−0.020*	0.067***	0.00300	−0.198***	−0.151***	1

*p < 0.1, **p < 0.05, ***p < 0.01.

Further, Table 3B shows the descriptive statistics of the main variables of enterprises with different ownership types. The average value of the corporate tax avoidance (TME) of state-owned enterprises is -0.034 while corporate tax avoidance (TME) of private enterprises is -0.019, indicating that overall, the degree of tax avoidance of state-owned enterprises is lower than the degree of tax avoidance of private enterprises. The mean value of management equity incentive (MRS) is 0.048 for SOEs and 0.479 for private firms, indicating that the degree of equity incentive for executives is higher in private firms than in SOEs.

Table 3C shows that the correlation coefficients among variables are either low or very weak and significant. From the sign of correlation coefficients among variables, the degree of corporate tax avoidance is positively correlated with the degree of executive equity incentive, which initially verifies the hypothesis of this paper. The correlation between enterprise size (SIZE), enterprise age (AGE), and tax avoidance is negative, while the correlation between enterprise growth capacity (GROWTH), non-interest tax shield (NDTS), enterprise value (TQ), the shareholding ratio of top shareholder (TOP1), and two jobs at once (DUAL) and tax avoidance is positive, which is essentially in line with the above inference.

### 4.2. Regression analysis

#### 4.2.1. Relationship between executive equity incentive and corporate tax avoidance

Table 4A shows the regression results of the effect of executive equity incentive on corporate tax avoidance when only the time effect is controlled. It is easy to spot that: (1) the regression coefficient of management equity incentive (MRS) and corporate tax avoidance (TME) is 0.017 without control variables, and it is significant at the 1% level, indicating that the degree of executive equity incentive has a greater impact on corporate tax avoidance. After adding the control variables, the sign of the regression coefficient between executive equity incentive and corporate tax avoidance is always positive and remains significant at least at the 5% level, thus it is inferred that there is a positive relationship between executive equity incentive and corporate tax avoidance, which verified Hypothesis 1 and consistent with prior studies (Phillips, 2003; Robinson et al., 2010; Rego and Wilson, 2012; Gaertner, 2014; Powers et al., 2016; Khan et al., 2019; Zhou and Hu, 2021).

**TABLE 4:** Regression results of the effect of executive equity incentive on corporate tax avoidance.

Variable	(1)	(2)	(3)
	TME	TME	TME
**Panel A: Controlling for time effects only**			
MRS	0.017***	0.013***	0.011**
	(3.99)	(2.72)	(2.12)
SIZE		−0.004***	−0.004***
		(-3.11)	(-2.61)
GROWTH		0.017***	0.017***
		(4.27)	(4.24)
NDTS		0.721***	0.715***
		(5.93)	(5.82)
TQ		0.006***	0.006***
		(6.28)	(6.38)
TOP1			0.023*
			(1.85)
DUAL			0.000
			(0.07)
AGE			−0.002
			(-1.04)
Constant	−0.055***	0.010	−0.000
	(-12.57)	(0.29)	(-0.01)
Year	Yes	Yes	Yes
*N*	12,274	12,274	12,274
*r* ^2^	0.007	0.016	0.016
*F*	16.122	21.915	16.740
**Panel B: Controlling for time and individual effects**			
MRS	0.029***	0.026**	0.024**
	(2.67)	(2.41)	(2.14)
SIZE		−0.027***	−0.026***
		(-3.60)	(-3.40)
GROWTH		0.020***	0.020***
		(4.46)	(4.47)
NDTS		−1.205***	−1.179***
		(-3.45)	(-3.31)
TQ		0.002	0.002
		(1.11)	(1.23)
TOP1			−0.001
			(-0.02)
DUAL			0.003
			(0.41)
AGE			−0.004
			(-0.50)
Constant	−0.053***	0.553***	0.552***
	(-9.21)	(3.34)	(3.26)
Year	Yes	Yes	Yes
Company	Yes	Yes	Yes
*N*	12,274	12,274	12,274
*r* ^2^	0.005	0.009	0.009
*F*	8.172	8.661	6.572

The *t*-statistics in parentheses, *p < 0.1, **p < 0.05, ***p < 0.01.

Table 4B indicates the regression outcomes while controlling for the time and individual effects. From the regression results, it can be concluded that without the control variables, controlling for the time and individual fixed effects, the relationship between the degree of management equity incentive (MRS) and the degree of corporate tax avoidance (TME) is still positive and significant at the 1% level is positively correlated with corporate tax avoidance (TME), and the *p*-value of corporate tax avoidance (TME) is less than 0.05, i.e., significant at the 5% level (in line with Liu et al., 2010; Armstrong et al., 2015; Zhang et al., 2019).

#### 4.2.2. Effect of internal control on the relationship between executive equity incentive and corporate tax avoidance

Table 5 shows the results of the regression based on Model 2. From the regression results, it can be shown that: (1) the coefficient of the interaction term MRS*IC between corporate executive equity incentive and the internal control index is 0.156 without controlling for time and individual effects, and it is significant at the 1% level, indicating that internal control plays an enhancing role in the positive relationship between corporate executive equity incentive and the degree of corporate tax avoidance (TME); (2) without controlling for time and individual effects, there is a significant positive connection between the interaction term MRS*IC and the degree of corporate tax avoidance (TME) after adding the control variables. (2) With the inclusion of control variables without controlling for time and individual effects, the interaction term between corporate executive equity incentive and internal control index (MRS*IC) and the degree of corporate tax avoidance (TME) has a significant positive relationship, which is significant at the 1% level. (3) Controlling for time effects only, the regression coefficient of the interaction term (MRS*IC) is 0.133 after adding the control variables, which is positively correlated with the degree of corporate tax avoidance (TME) and is significant at the 1% level (4) Controlling for individual effects, the regression coefficient of the interaction term (MRS*IC) is 0.130 after adding the control variables, which is positively correlated with the degree of corporate tax avoidance (TME). (5) After controlling for both individual and time-fixed effects, the regression coefficient of the interaction term (MRS*IC) is 0.123, which is positively correlated with the degree of corporate tax avoidance (TME), and is significant at the 5% level.

**TABLE 5:** The effect of internal control on the relationship between executive equity incentive and corporate tax avoidance.

Variable	(1)	(2)	(3)	(4)	(5)
	TME	TME	TME	TME	TME
MRS	0.011**	0.005	0.009	0.020*	0.020*
	(2.47)	(0.86)	(1.63)	(1.78)	(1.75)
IC	0.113***	0.124***	0.125***	0.106***	0.104***
	(7.69)	(8.27)	(8.32)	(5.28)	(5.16)
MRS*IC	0.156***	0.135***	0.133***	0.130**	0.123**
	(3.74)	(3.23)	(3.18)	(2.44)	(2.30)
SIZE		−0.006***	−0.006***	−0.015**	−0.029***
		(-4.02)	(-4.04)	(-2.07)	(-3.73)
GROWTH		0.012***	0.016***	0.013***	0.018***
		(3.03)	(3.96)	(2.90)	(3.97)
NDTS		0.774***	0.780***	−1.023***	−1.071***
		(6.34)	(6.41)	(-2.90)	(-3.04)
TQ		0.005***	0.006***	−0.000	0.001
		(5.47)	(6.05)	(-0.31)	(0.96)
TOP1		0.014	0.019	−0.024	−0.008
		(1.17)	(1.54)	(-0.53)	(-0.17)
DUAL		0.002	0.000	0.002	0.003
		(0.55)	(0.05)	(0.34)	(0.47)
AGE		−0.000	0.000	0.041***	−0.005
		(-0.15)	(0.09)	(4.24)	(-0.38)
Constant	−0.024***	0.084**	0.050	0.259*	0.615***
	(-14.35)	(2.46)	(1.46)	(1.65)	(3.67)
Year	No	No	Yes	No	Yes
Company	No	No	No	Yes	Yes
*N*	12,274	12,274	12,274	12,274	12,274
*r* ^2^	0.006	0.015	0.022	0.007	0.012
*F*	23.461	18.823	19.628	6.184	7.550

The *t*-statistics in parentheses, *p < 0.1, **p < 0.05, ***p < 0.01.

Overall, the coefficient of the interaction term MRS*IC between corporate executives’ equity incentive and internal control index is always positive and significant, indicating that internal control enhances the positive relationship between corporate executive equity incentive and the degree of corporate tax avoidance, which supports the previous hypothesis (in line with Henry et al., 2011; Balsam et al., 2014; Zhang et al., 2020).

#### 4.2.3. Effect of different ownership types on the relationship between executive equity incentive and corporate tax avoidance

Heterogeneity grouping tests on the nature of equity will be conducted next to illustrate the effect of different ownership natures of firms on the relationship between management equity incentive and corporate tax avoidance.

Table 6 shows the results of the grouped regressions derived from the nature of ownership. From the regression results, it shows that: (1) In state-controlled enterprises, controlling for both time and individual effects, the regression coefficient of management equity incentive (MRS) is 0.099, which is positively correlated with corporate tax avoidance (TME) and is significant at the 10% level (Zhang et al., 2019). After adding the control variables, the regression coefficient of management equity incentive (MRS) is 0.103, which is positively correlated with corporate tax avoidance (TME) and is significant at the 10% level. (2) Controlling for time effects, there is no significant correlation between management equity incentive (MRS) and corporate tax avoidance (TME) in private firms, and the same is not significant after adding the control variables. It is consistent with the previous hypothesis (i.e., consistent with Luo and Zeng, 2018; Zhang et al., 2019).

**TABLE 6:** Regression results of ownership nature grouping.

	State-owned enterprises (SOE)		Private enterprises	
	TME	TME	TME	TME
MRS	0.099*	0.103*	0.013	0.013
	(1.69)	(1.72)	(1.18)	(1.10)
SIZE		−0.034*		−0.021**
		(-1.94)		(-2.22)
GROWTH		0.017*		0.018***
		(1.85)		(3.41)
NDTS		−1.139*		−1.426***
		(-1.66)		(-3.21)
TQ		0.003		0.003
		(0.79)		(1.46)
TOP1		−0.144*		0.068
		(-1.69)		(1.04)
DUAL		−0.009		−0.001
		(-0.58)		(-0.10)
AGE		0.020		−0.003
		(0.51)		(-0.24)
Constant	−0.073***	0.736*	−0.036***	0.424**
	(-9.36)	(1.81)	(-4.27)	(2.02)
Year	Yes	Yes	Yes	Yes
Company	Yes	Yes	Yes	Yes
*N*	3988.000	3988.000	7189.000	7189.000
*r* ^2^	0.013	0.017	0.002	0.007
*F*	7.418	4.248	1.544	2.857

The *t*-statistics in parentheses, *p < 0.1, **p < 0.05, ***p < 0.01.

### 4.3. Robustness test

#### 4.3.1. Relationship between executive equity incentive and corporate tax avoidance

Table 7 displays the effects of the robustness test on the relationship between management equity incentive and corporate tax avoidance. According to the results, when the current effective tax rate is being used as a proxy for corporate tax avoidance, the degree of management equity incentive (MRS) and corporate tax avoidance (ETR) are inversely correlated and significant at the 1% level without controlling for the time and individual effects. The higher the degree of executive equity incentive, the lower the effective tax rate and the higher the degree of tax avoidance. As in the previous empirical study, the significance test reveals that there is a positive correlation between the extent of management equity incentive and the degree of business tax avoidance (Rego and Wilson, 2012; Gaertner, 2014; Khan et al., 2019; Zhou and Hu, 2021).

**TABLE 7:** Robustness test results of the relationship between executive equity incentive and corporate tax avoidance.

	(1)	(2)	(3)	(4)	(5)
	ETR	ETR	ETR	ETR	ETR
MRS	−0.062***	−0.030***	−0.035***	−0.028**	−0.029***
	(-14.41)	(-5.70)	(-6.56)	(-2.49)	(-2.59)
SIZE		0.010***	0.010***	0.013*	0.028***
		(6.28)	(6.32)	(1.74)	(3.63)
GROWTH		−0.011***	−0.016***	−0.016***	−0.021***
		(-2.78)	(-3.88)	(-3.63)	(-4.74)
NDTS		−0.883***	−0.891***	1.020***	1.111***
		(-7.14)	(-7.24)	(2.88)	(3.14)
TQ		−0.006***	−0.007***	0.000	−0.002
		(-6.36)	(-6.90)	(0.01)	(-1.14)
TOP1		0.013	0.008	0.030	0.009
		(1.05)	(0.62)	(0.68)	(0.19)
DUAL		0.001	0.003	−0.001	−0.002
		(0.22)	(0.78)	(-0.07)	(-0.22)
AGE		0.017***	0.016***	−0.024***	0.002
		(7.38)	(7.11)	(-3.42)	(0.27)
Constant	0.232***	−0.004	0.034	−0.041	−0.398**
	(103.60)	(-0.11)	(0.98)	(-0.26)	(-2.36)
Year	No	No	Yes	No	Yes
Company	No	No	No	Yes	Yes
*N*	12,274	12,274	12,274	12,274	12,274
*r* ^2^	0.017	0.039	0.048	0.004	0.008
*F*	207.508	62.163	50.807	4.084	6.179

The *t*-statistics in parentheses, *p < 0.1, **p < 0.05, ***p < 0.01.

#### 4.3.2. Moderating effect of internal control

The results of the robustness test on the effect of internal control on the relationship between executive equity incentive and corporate tax avoidance are shown in Table 8 and prove that, after using the current effective tax rate as a proxy for the degree of corporate tax avoidance, the degree of management equity incentive (MRS) is negatively related to the degree of corporate tax avoidance (ETR) without controlling for time and individual effects and is significant at the 1% level. Further, the interaction term (MRS*IC) is also negatively correlated with corporate tax avoidance (ETR) and is significant at the 5% level, indicating that internal control enhances the relationship between the degree of management equity incentive (MRS) and corporate tax avoidance (ETR); controlling for both time and individual effects, the interaction term between the degree of equity incentive and internal control index (MRS*IC) is negatively correlated and significant at the 5% level. As in the previous empirical study, the significance test again indicates that internal control has an enhanced effect on the relationship between the degree of executive equity incentive and the degree of corporate tax avoidance.

**TABLE 8:** Robustness test results of the moderating effect of internal control.

	(1)	(2)	(3)	(4)	(5)
	ETR	ETR	ETR	ETR	ETR
MRS	−0.058***	−0.026***	−0.031***	−0.025**	−0.024**
	(-13.46)	(-4.96)	(-5.85)	(-2.23)	(-2.18)
IC	−0.124***	−0.140***	−0.140***	−0.107***	−0.104***
	(-8.36)	(-9.28)	(-9.35)	(-5.34)	(-5.21)
MRS*IC	−0.127***	−0.092**	−0.089**	−0.129**	−0.124**
	(-3.01)	(-2.19)	(-2.14)	(-2.43)	(-2.32)
SIZE		0.013***	0.013***	0.018**	0.030***
		(7.95)	(8.02)	(2.46)	(3.94)
GROWTH		−0.010**	−0.015***	−0.014***	−0.019***
		(-2.54)	(-3.65)	(-3.26)	(-4.23)
NDTS		−0.952***	−0.959***	0.956***	1.001***
		(-7.78)	(-7.87)	(2.72)	(2.85)
TQ		−0.006***	−0.007***	0.000	−0.001
		(-6.06)	(-6.62)	(0.29)	(-0.86)
TOP1		0.019	0.014	0.030	0.016
		(1.56)	(1.14)	(0.68)	(0.35)
DUAL		0.001	0.003	−0.001	−0.002
		(0.25)	(0.82)	(-0.18)	(-0.29)
AGE		0.017***	0.016***	−0.039***	0.002
		(6.21)	(5.96)	(-4.12)	(0.19)
_cons	0.212***	−0.080**	−0.044	−0.136	−0.460***
	(124.52)	(-2.34)	(-1.28)	(-0.87)	(-2.76)
Year	No	No	Yes	No	Yes
Company	No	No	No	Yes	Yes
*N*	12,274	12,274	12,274	12,274	12,274
*r* ^2^	0.022	0.047	0.056	0.007	0.011
*F*	93.278	60.749	51.779	6.359	7.236

The *t*-statistics in parentheses, *p < 0.1, **p < 0.05, ***p < 0.01.

#### 4.3.3. Effect of ownership type

Table 9 shows the regression results for different ownership types after using the current effective tax rate as a proxy for corporate tax avoidance. Controlling for both time and individual effects, the regression coefficient of management equity incentive (MRS) of state-controlled enterprises is -0.097, which is negatively related with corporate tax avoidance (TME) and significant at the 10% level, while the regression coefficient of management equity incentive (MRS) of state-controlled enterprises is –0.103, which is negatively correlated with corporate tax avoidance (TME) and significant at the 10% level after adding the control variables.

**TABLE 9:** Robustness test results of the effect of ownership type on the relationship between executive equity incentive and corporate tax avoidance.

	(1)	(2)	(3)	(4)
	ETR	ETR	ETR	ETR
MRS	−0.097*	−0.103*	−0.016	−0.016
	(-1.67)	(-1.72)	(-1.43)	(-1.42)
SIZE		0.039**		0.022**
		(2.22)		(2.33)
GROWTH		−0.019**		−0.019***
		(-1.99)		(-3.66)
NDTS		1.044		1.361***
		(1.53)		(3.07)
TQ		−0.003		−0.004*
		(-0.82)		(-1.69)
TOP1		0.148*		−0.059
		(1.74)		(-0.90)
DUAL		0.012		0.001
		(0.74)		(0.10)
AGE		−0.023		0.000
		(-0.57)		(0.02)
_cons	0.281***	−0.631	0.210***	−0.266
	(36.24)	(-1.56)	(25.14)	(-1.27)
Year	Yes	Yes	Yes	Yes
Company	Yes	Yes	Yes	Yes
*N*	3988.000	3988.000	7189.000	7189.000
*r* ^2^	0.012	0.017	0.001	0.007
*F*	6.865	4.184	1.226	2.829

The *t*-statistics in parentheses, *p < 0.1, **p < 0.05, ***p < 0.01.

It demonstrates that the more the executive stock incentive, the lower the enterprise’s effective tax rate and the greater the degree of tax avoidance (consistent with Gaertner, 2014; Khan et al., 2019). In contrast, there is no significant correlation between management equity incentive (MRS) and corporate tax avoidance (TME) in private firms, and the same is not relevant after adding control variables. The robustness of the article results is confirmed, as is the case with the baseline regression results.

## 5. Conclusion and policy recommendations

The inconsistency of goals between shareholders and executives leads to a conflict of interests in the context of the separation of operation and ownership in modern enterprises and the principal-agent relationship between shareholders and managers. Implementing an equity incentive for corporate executives is considered a good way to alleviate the principal-agent problem, but it also raises the problem of corporate tax avoidance. This paper empirically analyzes the relationship between management equity incentive and corporate tax avoidance based on the literature on executive compensation incentives, internal control deficiencies and corporate tax avoidance at home and abroad (Phillips, 2003; Desai and Dharmapala, 2006; Robinson et al., 2010; Henry et al., 2011; Rego and Wilson, 2012; Balsam et al., 2014; Gaertner, 2014; Powers et al., 2016; Khan et al., 2019; Zhang et al., 2019, 2020; Zhou and Hu, 2021), by using A-share listed companies in China from 2016 to 2020 research sample. The results show that: (1) There is a positive relationship between management equity incentive and enterprise tax avoidance behavior. That is, the more the stock incentive offered to executives, the more likely corporations are to pursue tax avoidance strategies aggressively. When executives are given an equity incentive, their individual interests are linked with the company’s objectives, indicating that they are driven to enhance their business performance through tax avoidance by gaining bigger profits. (2) Internal control deficiencies enhance the positive correlation between equity incentive and enterprise tax avoidance behavior. According to objective statistics, most Chinese businesses are still in the early stages of their internal control development process. In Chinese businesses, the lack of an internal control system and the failure of internal control measures are prevalent, and such loopholes can intensify the tax avoidance behavior that arises when executives are subject to equity incentive. (3) The influence of management equity incentive on enterprise tax avoidance behavior is greater in state-owned firms than in private enterprises. State-owned enterprises have a greater likelihood of increasing enterprise tax avoidance behavior when management is subject to equity incentive for reasons such as strict performance requirements, lower regulatory oversight, and less interference from negative information.

Relevant managerial and policy implications are as follows: First of all, the state should require enterprises to fully disclose relevant information and reduce information asymmetry. It should increase the supervision as well as the punishment for enterprises’ wrongdoings to act as a deterrent for them. Simultaneously, the government should pay more attention to enterprises that have already committed tax irregularities and conduct regular inspections to prevent tax irregularities from happening again. Enterprises should fully recognize the important role of internal control system to their development, establish sound internal control and supervision mechanism, improve modern management system of enterprises and establish effective information communication mechanism. Moreover, for the issue of equity incentive of enterprises, the shareholding ratio of management should be strictly controlled and the equity structure should be optimized so that it cannot only achieve the purpose of incentive and improve the long-term economic interests of enterprises, but also consider the business risks brought by equity incentive to enterprises, reasonably control the risks and pay attention to the equity checks and balances of enterprises.

## Data availability statement

The data supporting the conclusions of this article will be made available by the authors.

## Author contributions

XW: conceptualization, methodology, and validation. MK: visualization, writing—original draft, and writing—review and editing. LQ: methodology, software, formal analysis, data curation, and writing—original draft. AR: validation and writing—review and editing. All authors read and approved the final manuscript.

## Footnotes

1
https://www.chyxx.com

2 Tax avoidance is defined as engaging in transactions and behaviors that reduce a firm’s tax burden (Dyreng et al., 2008).

3 For instance, a study from 2013 discloses that tech giant Apple utilized legal sleights to evade paying billions of dollars in U.S. taxes on $44 billion in overseas profits over the previous 4 years.

4 China Securities Net. White Paper on Internal Control of Listed Companies in China 2018 [EB/OL]. http://news.cnstock.com/news,yw-201807-4251023.htm,2018-07-26.
